# A Genomic Exploration of the Possible De‐Extirpation of the Zanzibar Leopard

**DOI:** 10.1111/mec.17566

**Published:** 2024-10-30

**Authors:** Xin Sun, Emily Louisa Cavill, Ashot Margaryan, Jianqing Lin, Søren Thingaard, Tamrini A. Said, Shyam Gopalakrishnan, M. Thomas P. Gilbert

**Affiliations:** ^1^ Center for Evolutionary Hologenomics, Globe Institute, Faculty of Health and Medical Sciences University of Copenhagen Copenhagen Denmark; ^2^ University Museum, NTNU Trondheim Norway; ^3^ Guangdong Provincial Key Laboratory of Marine Disaster Prediction and Prevention, Guangdong Provincial Key Laboratory of Marine Biotechnology, Institute of Marine Science Shantou University Shantou China; ^4^ Ferpharm Group, Hvedstrupvej 40 Hvedstrup Roskilde Denmark; ^5^ Department of Forestry Zanzibar Tanzania

**Keywords:** conservation genetics, inbreeding, mammals, phylogeography, population genetics, wildlife management, Zanzibar leopard

## Abstract

The recently extirpated Zanzibar leopard was the only known African leopard (*Panthera pardus* spp.) population restricted exclusively to a major island habitat. Although its demise was driven through habitat change and conflict with humans, given its role as a keystone species for the Zanzibar Archipelago, its successful potential reintroduction might offer a means for helping preserve the natural biodiversity of its former habitat. Whether this is feasible, however, would be contingent on both whether closely related source populations can be identified on mainland Africa, and whether the Zanzibar form exhibited any special adaptations that might need to be considered when choosing such a source. In light of these questions, we genomically profiled two of the six known historic specimens, to explore whether they represent a realistic candidate for de‐extirpation through reintroduction. Our analyses indicate that despite its geographical separation, the Zanzibar leopard shared a close genetic relationship with mainland East African individuals. Furthermore, although its uniqueness as an island population was emphasised by genomic signatures of high inbreeding and increased mutation load, the latter similar to the level of the critically endangered Amur leopard (*P. p. orientalis*), we find no evidence of functionally significant genetic diversity unique to Zanzibar. We therefore conclude that should attempts to restore leopards to Zanzibar be considered, then mainland East African leopards would provide a suitable gene pool.

## 1 Introduction

Biodiversity losses resulting in both the extirpation of unique populations, and extinction of whole species, have intensified in past decades despite the increase of conservation efforts (Butchart et al. 2010; Pereira et al. 2010; Seddon et al. 2014). To restore lost diversity and ecosystem functions, the validity of a range of different conservation approaches are currently being both debated, and implemented (Fernandez et al. 2017; Perino et al. 2019; Seddon et al. 2014; Svenning et al. 2016). For example, given recent technological advances in genome editing and the retrieval of genetic information from Pleistocene megafauna (Dabney et al. 2013; Gansauge and Meyer 2013), the heavily debated idea of de‐extinction as a creator of proxy for lost biodiversity in order to restore ecological function is gaining traction (IUCN/SSC 2016; Lin et al. 2022; Novak 2018; Richmond, Sinding, and Gilbert 2016; Seddon, Moehrenschlager, and Ewen 2014; Shapiro 2017). On the other hand, technologically simpler methods are also being explored, including back‐breeding to restore lost phenotypes or even lost species (The Quagga Project n.d.; The Tauros Programme n.d.), as well as reintroduction of extirpated populations from closely related gene pools (Lorimer et al. 2015).

To date, relatively few successful examples have been delivered using these approaches. Of these, reintroduction approaches have delivered the most success in recent years. These include species that span diverse spatial, temporal and genetic backgrounds (Germano and Bishop 2009; Griffiths et al. 2013; Keedwell, Maloney, and Murray 2002). For example, the rewilding of the wolf population in the Yellowstone National Park has played the role of a keystone species (Lorimer et al. 2015; Ripple and Beschta 2012) and reshaped the local ecosystem. And the replacement of an extinct, with a related extant, tortoise species has been used to fill an otherwise vacant niche in the Galapagos Islands (Hunter et al. 2013; Lorimer et al. 2015). In a different context, back‐breeding has been used to generate morphologically (if not genomically) similar quagga‐like plain zebras, as a result of the selective crossing of closely related zebra subspecies (Larison et al. 2021; The Quagga Project n.d.). And lastly, although still in its early stages, genome editing technologies such as CRISPR‐Cas9, have boosted a growing interest in ambitious projects that aim to resurrect now lost megafauna, such as the woolly mammoth (Colossal/A New Dawn of Genetics n.d.), and Tasmanian tiger (Colossal/A New Dawn of Genetics n.d.).

Ultimately, for any of these approaches to gain favour, test cases are needed to explore whether the reality matches the expectations. In this regard, it could be argued that ideal candidates are those that (i) have perceived conservation benefits, (ii) are technically feasible, and (iii) likely to generate sufficient support from the public (IUCN/SSC 2016; Seddon, Moehrenschlager, and Ewen 2014). Furthermore, in addition to the technical limitations associated with resurrection attempts (e.g., relating to integrity of the candidate genome), analyses of risks for possible impacts on both the ecology and human society of the region that would receive the reintroduced or resurrected species, need to be performed (Iacona et al. 2017; Seddon, Moehrenschlager, and Ewen 2014).

As a thought experiment, we speculated that the Zanzibar leopard could offer an interesting candidate for de‐extirpation through reintroduction. As a morphologically distinct population of a charismatic big cat species (the leopard [*Panthera pardus*]), it belongs to a species that exhibits extraordinary adaptation to diverse habitats, something critical should animals require introduction to novel habitats. Furthermore, in comparison to other big cats, surviving African leopards maintain relatively high genomic diversity, coupled to low levels of differentiation (Paijmans et al. 2021; Pečnerová et al. 2021). Unfortunately however, while once endemic to Unguja Island, ultimately it failed to survive the challenge of human colonisation (Walsh and Goldman 2007), likely being fully extirpated by the end of the 20th century (Goldman and Walsh 2002) due to extensive hunting. With regards to phenotype, in comparison to its continental relative, this apex predator is thought to have had a smaller body size and distinct coat pattern (Pakenham 1984). With no captive individuals known, six preserved specimens collected in the early 20th remain the only source from which to study this population (Walsh and Goldman 2008).

## 2 Materials and Methods

### 2.1 Sample Collection, DNA Extraction and Sequencing

Samples were collected from three of the six known preserved Zanzibar leopard specimens: one from the Zanzibar Museum specimen provided under permit IMMK/MKM/68/VOL.IV/36 (sample ID Z 1209, that dates to the first half of the 20th century (Walsh and Goldman 2008)), and one from each of the two specimens held at the Harvard Museum of Comparative Zoology under permit 2020‐1‐Cryo (sample IDs MCZ36709, 1937 and MCZ40953, 1939).

DNA was extracted in an aDNA‐dedicated PCR‐free laboratory, using aDNA methods following (Ramos‐Madrigal et al. 2021). We were unable to construct useable libraries from sample Z 1209, thus only MCZ36709 and MCZ40953 were successfully converted into BGISeq‐compatible genomic libraries using a tailored version of the Santa Cruz Reaction (SCR) (Kapp, Green, and Shapiro 2021; van Grouw et al. 2023) single‐strand library construction protocol. Libraries were purified using Qiagen MinElute clean‐up columns. Four 3‐μL replicates from each library template were amplified, using 2.5 U PFU Turbo CX Polymerase, 1× PFU Turbo buffer, 0.4 mg ml^−1^ bovine serum albumin (BSA), 0.25 μM mixed dNTPs, 0.1 μM BGI forward primer, 0.1 μM BGI reverse index‐primer per 50 μL reaction. Initial denaturation of libraries was carried out at 95°C for 2 min, followed by 30 cycles of denaturation at 95°C for 30 s, annealing at 60°C for 30 s, and extension at 72°C for 110 s, and the final extension held at 72°C for 10 min. PCR products were then purified with MagBio HiPrep PCR clean‐up beads. Amplified libraries were initially sequenced on the DNBSEQ‐G400 platform using BGI Copenhagen's commercial service. In order to generate a high coverage genome for this species, we selected the sample with the highest endogenous DNA content for deeper sequencing (MCZ36709). Subsequently, five additional Illumina libraries were built following the SCR protocol. Three 5μL PCR amplification replicates (following the PFU protocol as above, using Illumina indexes, and 16 thermal cycles per replicate) were made from each of the five new library templates. These were pooled and sequenced across one Illumina Novaseq S4 lane.

### 2.2 Data Mapping and Dataset Preparation

We used the *Paleomix* v1.2.13.2 (Schubert et al. 2014) pipeline to map the Zanzibar leopard sequencing data, and data from 79 previously published leopards (Paijmans et al. 2021; Pečnerová et al. 2021), against the domestic cat (*Felis catus*) reference genome (felCat 9.0, RefSeq assembly accession: GCF_000181335.3).

For quality control, we first estimated the error rate using an outgroup (felCat 9.0) and an error‐free individual (Amurleopard_PPO1) (Orlando et al. 2013) using ANGSD. Then, we estimated sample relatedness using ngsRelate (Hanghøj et al. 2019). A total of 21 samples were removed due to high error rate, or because they shared a close relatedness (duplicate of a specimen, or first‐degree relationship) with one sample in our dataset.

Genotype likelihoods were estimated with ANGSD (Korneliussen, Albrechtsen, and Nielsen 2014). We used the GATK model (−GL 2), and used reads with mapping quality above 30 (−minMapQ 30) and bases with sequencing quality above 20 (−minQ 20). Sites with a minor allele frequency of less than 0.05, or with more than 50% of the samples having missing genotype information, were removed. In order to minimise the effect of aDNA‐induced errors in the data generated from the historical sample, we removed transitions from all subsequent analyses. This resulted in a genotype likelihood panel containing 6,647,110 sites, that we refer to henceforth as the "genotype likelihood panel". The genotype likelihood panel was used for PCA, NGSadmix and phylogenetic analysis.

We also generated a pseudo‐haploid panel using ANGSD, by randomly sampling one read (−dofasta 1). Only samples that passed error rate filtering, with relatedness level above 1st degree and a minimum sequencing depth above 5×, were used (*N* = 38, Table S1) to determine the variable sites. The following command line was used to generate the pseudo‐haploid fasta sequence for each sample: –dofasta 1 –doCounts 1 –minQ 20 –minmapq 20 –setminDepthInd 1 –remove_bads 1 –uniqueOnly 1. The variable site panel was then converted to Plink files for further filtering using Plink v2.0 (Chang et al. 2015), and filtered to remove transitions, variants annotated as repeat sequence regions, and singletons. This resulted in a pseudo‐haploid panel with 2,709,567 sites. We then called pseudo‐haploid genotypes on all samples that passed quality control (*N* = 60), and refer to these as the "pseudo‐haploid panel". The pseudo‐haploid panel was used for f‐statistics and qpAdm analysis.

We called genotypes for samples that had a sequencing depth above 10×, and that passed the quality control. Samtools v1.4 (Li et al. 2009) was used for genotype calling with the following parameters *‐Q 20 ‐q 20 ‐B ‐‐ff 260*. Only biallelic SNPs were kept. To reduce genotype calling error, sites with sequencing depths of lower than 5×, and heterozygous genotypes with sequencing depths for each allele of less than 3×, were masked as missing. The genotype panel was used for runs of homozygosity, genetic load analysis, and gene ontology analysis.

### 2.3 Population Structure With PCA and NGSadmix

Principal component analysis (PCA) was performed using PCAngsd (Meisner and Albrechtsen 2018) on the genotype likelihood panel. We conducted two separate PCA analyses, one with all 60 samples that passed the quality control and relatedness filter, and another with only the 50 African leopards that had also passed the same filtering criteria (Table S1). To infer population structure and admixture for each sample, we used NGSadmix (Skotte, Korneliussen, and Albrechtsen 2013) with the genotype likelihood panel of 60 samples as input (Table S1). We ran the analysis assuming two to six ancestral populations. Each run was performed independently, with 100 replicates, and checked manually to assure convergence of the result.

### 2.4 Genetic Affinity With Outgroup f3‐Statistics and IBD Inference

To obtain the shared ancestry between the Zanzibar and other African leopards, we calculated outgroup *f3*‐statistics. The jaguar (Figueiró et al. 2017) was used as the outgroup, and the shared ancestry was measured since the separation between the Zanzibar leopard and other African leopards. *f3*‐statistics were calculated using admixtools2 (Maier et al. 2023; Patterson et al. 2012) with the haploid panel. We also used LocalNgsRelate (Severson, Korneliussen, and Moltke 2021) to infer identity‐by‐descent (IBD) sharing along the genome between the Zanzibar and other African leopards, using default parameters.

### 2.5 qpAdm Modelling of Zanzibar Leopard Ancestry

To model the ancestry of the Zanzibar leopard, we used qpAdm (Harney et al. 2021; Patterson et al. 2012) to explore the best possible scenario. Following our population structure results, we selected the following samples to represent major African leopard ancestry: Tanzania (4343_Tanzania), Zambia (ZMUC4446), Namibia (7942_Namibia), NorthEast Africa (SMNH595313) and NorthWest Africa (SMNH582373). qpAdm was run with admixtools2 (Maier et al. 2023; Patterson et al. 2012). For each sample, the models were considered feasible with a p‐value threshold lower than 0.01 as compared to the full model, and we then selected the model with the minimum amount of source populations as the best fitted model. In our case, our high coverage Zanzibar leopard (MCZ36709) sample rejected a single ancestry source model, with the best fitted model indicating two ancestry components.

### 2.6 Heterozygosity, Runs of Homozygosity and Genetic Load Estimation

We estimated heterozygosity for each sample using ANGSD (Korneliussen, Albrechtsen, and Nielsen 2014). The folded site frequency spectrum (SFS) was first estimated using the domestic cat reference genome as the ancestral state. Next heterozygosity was calculated based on the SFS. We excluded transitions for the SFS estimation. Only samples that had sequence depth above 10×, and that passed the quality control thresholds, were included in this analysis. To infer runs of homozygosity within the leopard genomes, we used Plink v1.9 (Chang et al. 2015) with the following settings to adjust for the likely short ancient DNA fragments, and to allow for more missing in each ROH region. The detailed parameters were *‐‐homozyg‐snp 30 ‐‐homozyg‐kb 500 ‐‐homozyg‐density 30 ‐‐homozyg‐gap 1000 ‐‐homozyg‐window‐snp 30 ‐‐homozyg‐window‐het 1 ‐‐homozyg‐window‐missing 10 ‐‐homozyg‐window‐threshold 0.05*.

We estimated genetic load using several different approaches. As load can be quantified by measuring the accumulation of deleterious mutations in the genome, we identified deleterious mutations with two different approaches. The first considers deleterious mutations as homozygous derived mutations in leopards, using conservation scores based on multi‐species alignment. PhyloP and PhastCons scores based on 100 vertebrate species were used (Hubisz, Pollard, and Siepel 2011). The conservation scores were stored with reference to the hg38 human reference genome. We retained the loci with the top 5 percentage scores, and mapped the coordinates in hg38 to felcat9 using *liftover*. The domestic cat reference genome was used as the ancestral state for the leopard genomes. Genetic load was calculated as the sum of the conservation score of homozygous derived loci in each individual. In this analysis, to reduce the effect of ancient DNA miscoding lesions, we excluded transitions. The second method considered deleterious mutations as mutations that have functional effects on coding regions. To determine these, we annotated variable sites in leopards using SnpEff v5.1 (Cingolani et al. 2012). Next, genetic load was quantified for each individual as the total count of homozygous derived loss of function and nonsynonymous mutations/synonymous mutations.

### 2.7 Gene Ontology for Genes With Homozygous Derived Mutations Specific to the Zanzibar Leopard

We generated the gene list containing homozygous derived mutations specific to the Zanzibar leopard sample. GO term and KEGG pathway enrichment analyses were performed with the KOBAS 3.0 web server (Bu et al. 2021). A threshold of *p* value < 0.05 after multiple testing corrections via FDR estimation was considered significant.

## 3 Results

### 3.1 Genome Resequencing of the Zanzibar Leopard

**FIGURE 1:**
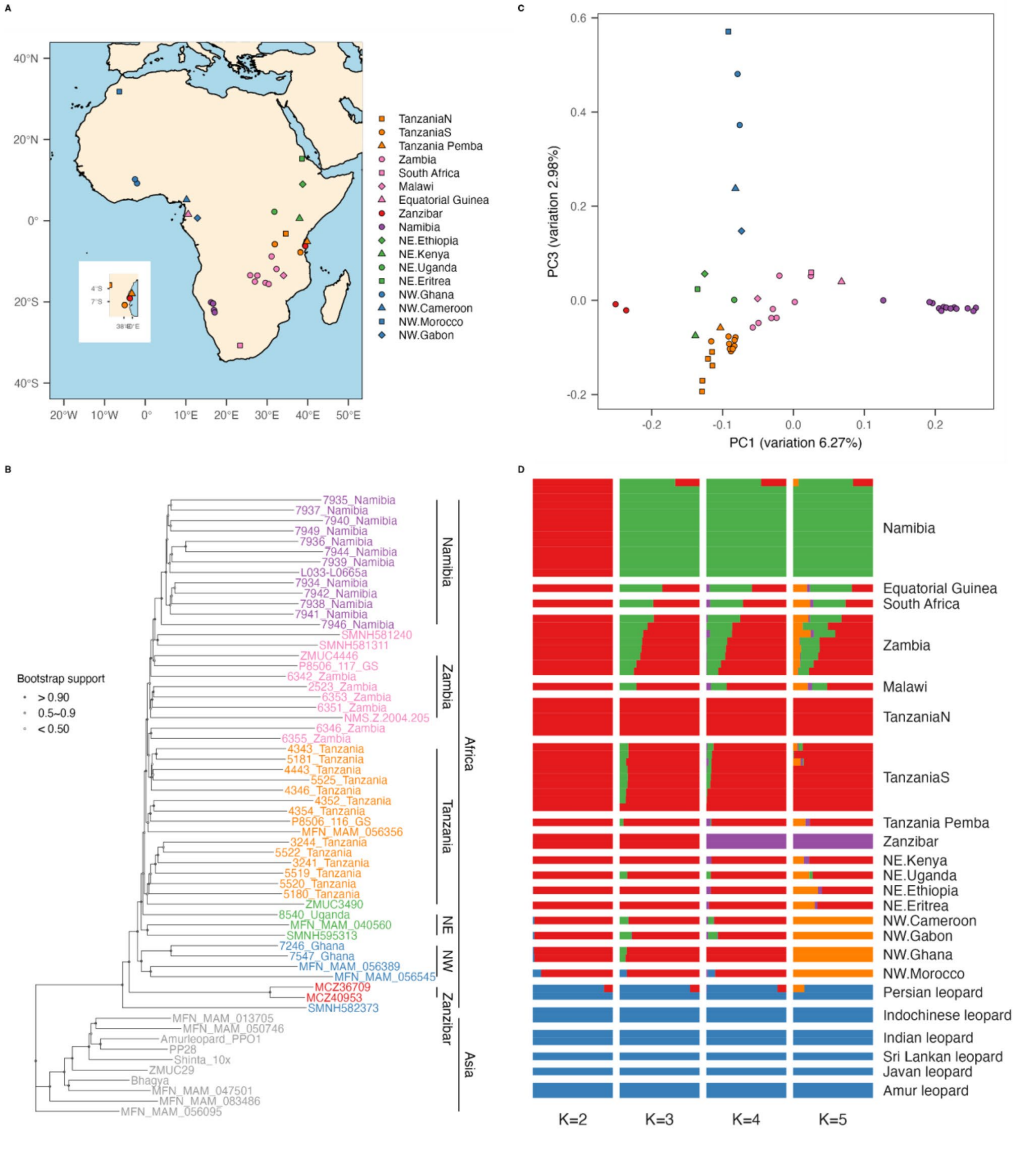
Population structure of African leopards. (A) Geographical origin of the Zanzibar leopard and other African leopard samples (Table S1). (B) Whole‐genome phylogeny inferred using Neighbour‐Joining methods. Bootstrap support values are shown by node colours. (C) Principal component analysis portraying the genomic diversity of African leopards. Samples are labelled with their geographical origin. Icons in A–C were coloured based on their geographical proximity. (D) ngsAdmix plot assuming two to five different ancestries of Asian and African leopards.

Three of the six known Zanzibar leopard specimens (Walsh and Goldman 2008) were sampled for this study. These include a sample of hairs from the specimen held on display at the Zanzibar Museum (Z 1209) that dates to the first half of the 20th century (Goldman and Walsh 2002), and cuttings of skin and dried tissue from both specimens held at the Harvard Museum of Comparative Zoology (MCZ36709, 1937 and MCZ40953, 1939). DNA was extracted and sequenced from all three samples. The sample from the Zanzibar museum specimen failed to deliver any usable sequencing libraries, thus was discarded from further analysis. In contrast, both Harvard specimens yielded rich libraries that contained relatively high levels of endogenous DNA (70.7% for MCZ36709 and 38.8% for MCZ40953). Given both the similarity in age of the two specimens, and the results of an initial analysis revealing they carried nearly identical mitogenome sequences (Figure S1), we elected to focus subsequent sequencing on the sample with best quality DNA (MCZ36709). This resulted in final average genomic depth of coverage of 38.4×. Next, to clarify the genetic ancestry of the Zanzibar leopard, a genomic dataset representing the genetic background of modern and historic leopards (average sequencing depth of 9×) was assembled from published studies (Paijmans et al. 2021; Pečnerová et al. 2021). Subsequent analyses were restricted to transversions, to account for possible DNA damage derived errors in the resulting dataset. This yielded a final matrix containing genotype likelihoods for 6,647,110 transversions, representing 60 African and Asian leopard samples (Figure 1A, Table S1).

### 3.2 The Genetic Affinity of the Zanzibar Leopard

To explore the genetic affinity between the Zanzibar and other leopards, we first looked at the population structure of our dataset. The results (Figure 1B–D and Figure S2) both recapitulate previous observations of continent level separation in leopards (Paijmans et al. 2021; Pečnerová et al. 2021), and reveal that the Zanzibar leopard falls within the diversity of African leopards. Subsequently, we restricted PCA analysis to African leopards, and observed that the placement of the Zanzibar leopards is distinct from the major Eastern and Southern population clusters of Zambia, Namibia, and Tanzania (Figure 1C) in principal components (PC) 1 and 3, while PC2 captures a high amount of drift in the Zanzibar leopard in comparison to other African leopards (Figure S3). Additional analysis in a whole‐genome phylogeny also revealed the distinctiveness of the Zanzibar leopard, with both sequenceable Zanzibar leopards placed close to the basal branch of African leopards, next to the isolated Morocco sample (Figure 1B). We highlight however, that although several distinct clusters form within the African leopards in the phylogeny, the node support for each clade was not high. This is possibly due to gene flow between these populations, and/or incomplete lineage sorting due to a recent divergence time (Pečnerová et al. 2021). Extra support for the unique placement of the Zanzibar leopard occurs when estimating the individual admixture proportions for each leopard sample at *K* = 4, as the Zanzibar leopard formed a distinct cluster (Figure 1D, Figure S4).

### 3.3 Low Levels of Divergence Between the Mainland and Island Population

**FIGURE 2:**
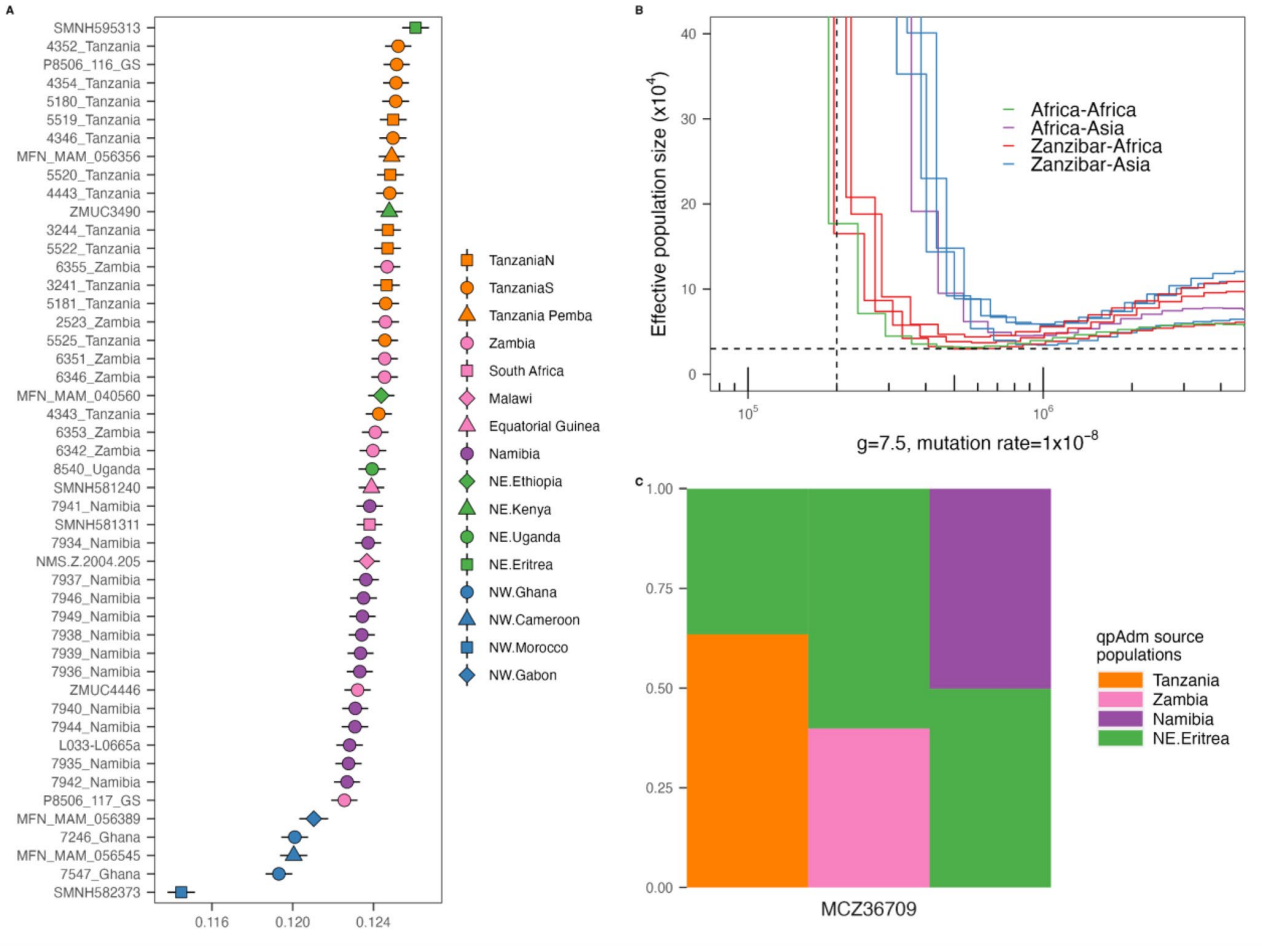
Genetic affinity between the Zanzibar and mainland African leopards. (A) Outgroup *f*3‐statistics comparing shared ancestry between the Zanzibar leopard and mainland African leopards. Icons are coloured and shaped according to their geographical and genetic clusters. (B) Divergence dating by hPSMC showing a similar level of divergence between the Zanzibar leopards and mainland African leopards, and within mainland African leopards. A pseudo‐diploid X chromosome of two male leopards excluding transition sites was used, assuming a generation time of leopards as 7.5 years (Pečnerová et al. 2021) and the mutation rate as 1 × 10^−8^ per nucleotide per generation (Figueiró et al. 2017). (C) qpAdm modelling of the ancestry components of the Zanzibar leopard. All best fitted models (*p* value < 0.01 and two source populations) are shown.

As the Zanzibar leopard is believed to be extinct in the wild, and because no reliable records exist of captive individuals (Goldman and Walsh 2002), the gene pool of mainland African leopards would be the most suitable source for any future re‐introduction attempt. We explored this by asking which mainland African population has the closest relationship with the Zanzibar leopard. Specifically, outgroup *f*3‐statistics were used to compare the shared ancestry between the Zanzibar and other leopards (Figure 2A). Despite the isolated structure revealed in the PCA and phylogeny, most ancestry was shared with samples from the geographically proximate populations, including those from NorthEast Africa (Eritrea), and Tanzania. Also notable was the observation that a previously sequenced sample labelled as originating from Pemba Island within the Zanzibar Archipelago, is more closely related to the mainland Tanzanian samples than our Zanzibar specimen (Figure S5). Given that a previous study has questioned the reliability of the geographical origin assigned to the Pemba Island sample (Paijmans et al. 2021), we cannot rule out that it may truly derive from the African continent. To obtain a more comprehensive understanding of its ancestry, we then modelled the Zanzibar leopard ancestry using major African leopard populations. The best fitted models for the high coverage Zanzibar leopard sample (MCZ36709) revealed a dominant ancestry component related to the North‐Eastern and Tanzanian population (Figure 2C).

To further investigate the relationship between the Zanzibar and other leopards, we estimated their relative divergence time using hPSMC (Figure 2B). To do this, hybrid leopard X chromosomes were generated, and compared between pairs of male individuals. These included (i) the Zanzibar leopard and one other African leopard, (ii) the Zanzibar leopard and one other Asian leopard, (iii) two randomly selected African leopards to represent within Africa divergence and (iv) one African leopard and one Asian leopard to represent inter‐continental divergence. Our analyses reveal that the estimated divergence time between the Zanzibar and other African leopards is similar to the divergence time within the mainland African leopards, ca 200,000 years ago. Although the Zanzibar leopard and the mainland African leopards shared similar divergence times, we identified that an extremely low number (< 0.005) of identical‐by‐descent (IBD) segments are shared between the Zanzibar leopard and its mainland relatives. This observation would be consistent with a lack of recent gene flow between the two groups (Figure S6).

### 3.4 Low Genetic Fitness and Lack of Unique Genomic Adaptation Signals to the Island

**FIGURE 3:**
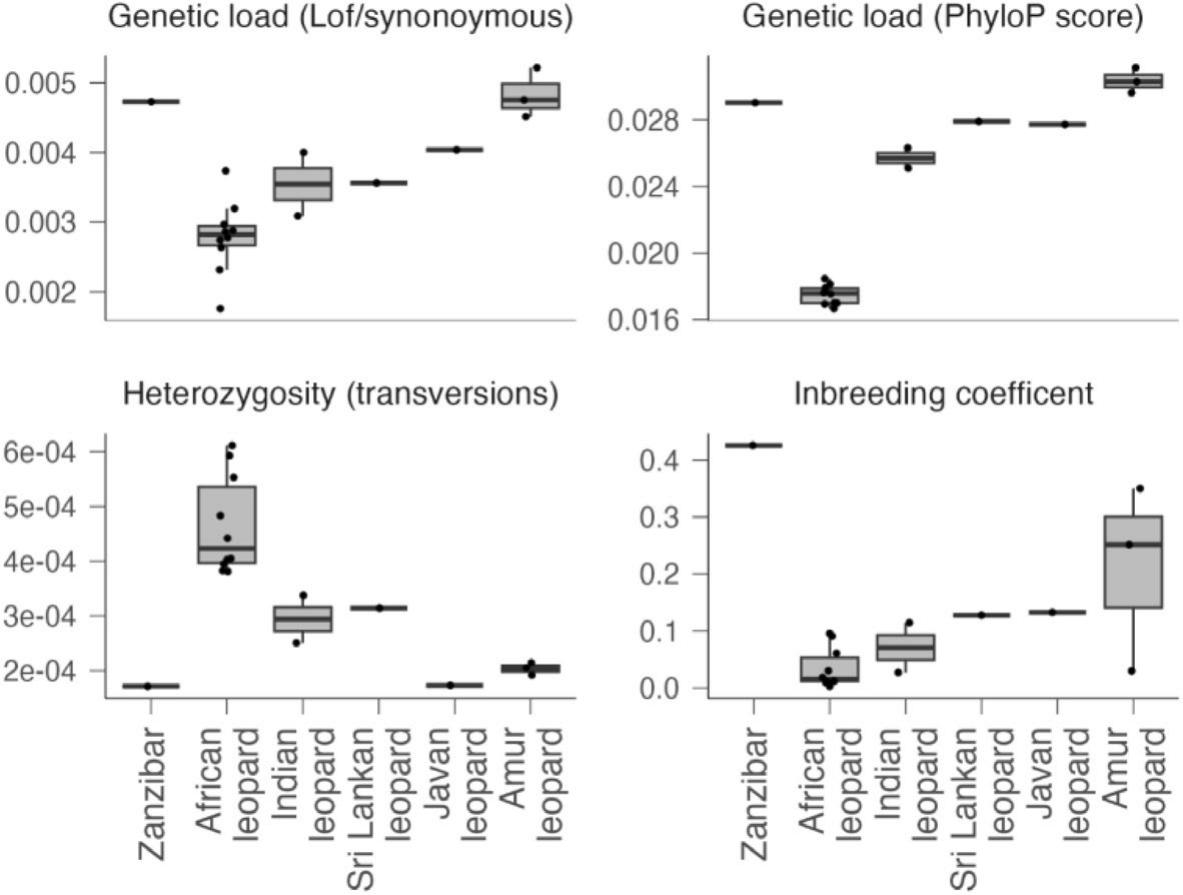
Genome‐wide heterozygosity, inbreeding coefficient (F_ROH_), and genetic load for different leopards. Samples are grouped by different leopard subspecies, with the Zanzibar and the NorthEast African population shown separately. Heterozygosity was calculated for each sample from the SFS estimated by ANGSD. Inbreeding coefficients (F_ROH_) were calculated as the proportion of the genome covered by long runs of homozygosity (ROH ≥ 1 Mb). Genetic load was calculated as both the sum of high PhyloP scores (top 5 percent across the whole‐genome) of homozygous derived transversion sites, and the ratio of loss of function(LoF)/synonymous homozygous derived transversion sites.

In light of its close genetic relationship with the North‐Eastern and Tanzania population, we hypothesise that the apparent morphological uniqueness of the Zanzibar leopard resulted from its existence as an island population. In general, island mammal populations tend to exhibit less diversity than mainland populations (Frankham 1997). We investigated whether this was the case for the Zanzibar leopard, by comparing the level of genetic fitness‐related parameters in the Zanzibar versus other leopards (Figure 3). In comparison to other African leopards, the Zanzibar leopard showed a much lower level of individual genome‐wide heterozygosity, at about one‐third of the level of its mainland neighbours. A higher inbreeding coefficient estimated from long runs of homozygosity was also estimated for the Zanzibar leopard, with over 40% of its genome lacking heterozygosity. This in turn gave it an increased mutation load. Overall therefore, the genetic fitness of the Zanzibar leopard was comparable to the currently critically endangered Amur leopards (*P. p. orientalis*) (Paijmans et al. 2021; Uphyrkina et al. 2002). However it is striking that this was already the case in the Zanzibar leopard at time of collection in the 1930s, something that might have been caused by long term conflicts with humans within the geographically restricted area of Unguja island (Goldman and Walsh 2002).

To explore for any possible effects of increased mutation load, we annotated the Gene Ontology and KEGG pathways of all the genes that exhibited Zanzibar leopard specific increased levels of homozygous derived loss of function and non‐synonymous alleles. Only one pathway, ‘bile secretion’ (FDR adjusted *p* value 0.037), was found to be significantly enriched in this list (Table S2). Although we speculate that this could be related to the difference in food resources between the Zanzibar archipelago and mainland Africa, we caution both that only one individual was included in this analysis, and that leopards are known to have a broad dietary niche (Athreya et al. 2016; Havmøller et al. 2021). We additionally explored for a possible genetic basis for the Zanzibar leopard's reduced body size and unique coat patterns (Table S3). Our results identified that a number of genes related to body size (Chase et al. 2002; Hayward et al. 2016; Hoopes et al. 2012; Jones et al. 2008; Parker et al. 2009; Plassais et al. 2019, 2022; Rimbault et al. 2013; Sutter et al. 2007; Vaysse et al. 2011), such as *SMOC2* and *LCORL*, and coat colouration patterns (*MC1R*) (Eizirik et al. 2003, 2010; Kaelin, McGowan, and Barsh 2021; Peterschmitt et al. 2009) exhibit homozygous non‐synonymous alleles in the Zanzibar sample (Table S4). Interestingly, while these may explain the phenotype, we note that none of them are unique to the Zanzibar leopard population, but rather vary in allele frequency in the mainland African leopards. This is information that could be relevant to consider if attempts were to be made to restore the lost phenotype.

## 4 Discussion

### 4.1 Re‐Introduction as a Means to De‐Extirpate the Zanzibar Leopard

In light of our results, we return to the thought experiment that initiated this work, namely whether the Zanzibar leopard could hypothetically represent an interesting candidate for possible de‐extirpation on Unjuga Island. While we wish to make it clear that we are unaware of any current exploration of the feasibility of such a project, we feel our results at least provide some insights into how it could be done, if the local authorities wished to pursue the idea further. On the one hand, our results suggest that a straightforward solution could be the translocation of mainland African leopards from NorthEast Africa onto the islands. On the other hand, more sophisticated solutions could also be considered, aimed at generating a leopard that more closely resembled the original form, using the genomic information generated in our study. In this context, currently three principal methods exist: back‐breeding, cloning, and genetic engineering (Richmond, Sinding, and Gilbert 2016; Shapiro 2015). Back‐breeding approaches involve, at the simplest level, selective breeding to reacquire the lost target's phenotypic traits, as has been pioneered in the context of the quagga (*Equus quagga*) (The Quagga Project n.d.) and the aurochs (*Bos primigenius*) (Stokstad 2015; The Tauros Programme n.d.). Therefore, attempts could be made to breed for the reduced size and unique coat morphology using carefully chosen morphological variants found on mainland Africa. At a more advanced level, should extant relatives retain partial genomic fragments derived from the lost form (as has been for example reported in some modern cattle breeds for the aurochs (Park et al. 2015)), then a more nuanced form of back‐breeding could be attempted. Specifically, living relatives chosen based on their genomes containing different genomic tracts derived from the lost form could be crossed, ultimately deriving offspring that contain incrementally more of the lost genome (Sinding and Gilbert 2016). Unfortunately however, given we found no evidence of significant IBD tracts shared between the Zanzibar and mainland African leopards, this does not seem a feasible solution. The second general approach, cloning, is unfortunately not an option, given that no known living Zanzibar leopards exist from which to source the viable cells needed. The final option, genetic engineering, will become increasingly interesting as the technical challenges that it currently faces are resolved (Colossal/A New Dawn of Genetics n.d.). Such an approach could in theory allow direct editing of genomes derived from extant continental African leopards with multiple aims. First, to introduce genetic variants identified in the Zanzibar leopard genome that are believed to be relevant for its morphology and inhabiting its niche. However we caveat that for this to be valid, one would have to also assume that its habitat has not changed so much, that it would render them maladaptive in the present. And secondly, and perhaps more likely in the long run to have a positive effect, to ensure that sufficient genetic diversity exists within the recreated population, to enable them to maintain a genomically healthy population.

In summary, deep sequencing of a Zanzibar leopard genome has enabled us to resolve its genetic ancestry, and in doing so provide suggestions for possible routes to its de‐extirpation, should the local community on Zanzibar one day wish to explore this option. Naturally, an obvious caveat is that as so few specimens remain for study, our analyses may not reveal a fully representative insight. However, we feel this is unlikely considering the levels of inbreeding observed.

## Author Contributions

M.T.P.G., S.T., T.A.S. and X.S. conceived the study. E.L.C. performed the ancient DNA lab work. X.S., A.M., E.L.C. and J.Q.L. performed bioinformatic analysis with input from S.G. and M.T.P.G. S.T. and T.A.S. provided samples from the Zanzibar museum. X.S. wrote the manuscript with input from all authors.

## Conflicts of Interest

The authors declare no conflicts of interest.

## Supporting information


Data S1.

Table S1.

## Data Availability

Whole‐genome re‐sequencing data are deposited at NCBI GenBank under the accession number of PRJNA892480. The detailed data processing pipeline for this project is available at https://github.com/xin‐sun‐popgen/zanzibar_leopard.
