# Supplementary material for: A Genomic Exploration of the Possible De‐Extirpation of the Zanzibar Leopard
Source: Mol Ecol. 2024 Oct 30;34(23):e17566. doi: 10.1111/mec.17566 (PMC12684304; doi:10.1111/mec.17566)
Supplement: Supplementary file 1 — Data S1. [file MEC-34-e17566-s002.docx]

# Supplementary Figures


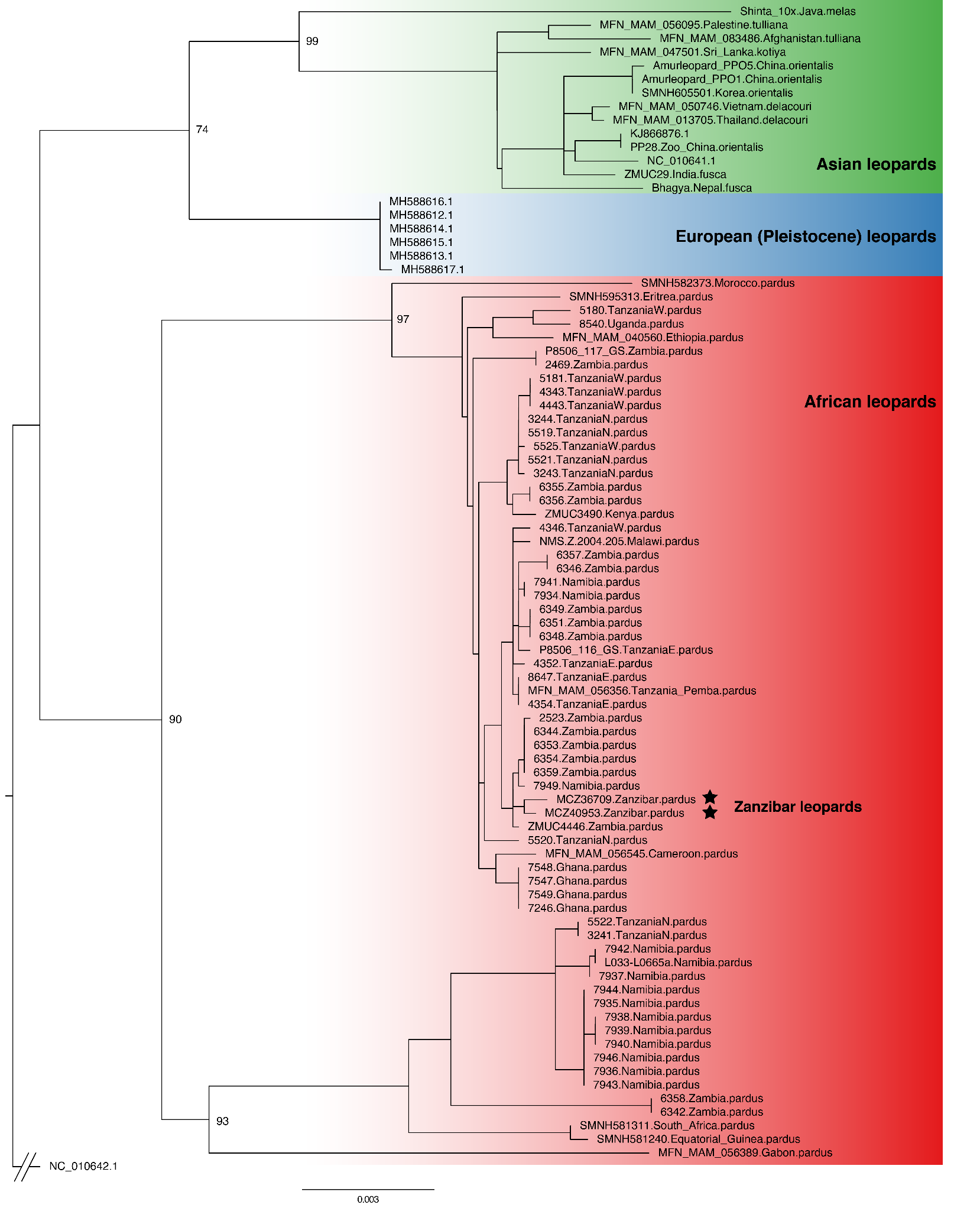


Fig S1. Maximum likelihood phylogeny of mitochondrial sequences. Regions containing potential *numts* were excluded. Bootstrap support was labelled for major clades. The tiger mitochondrial sequence was used to root the phylogenetic tree.


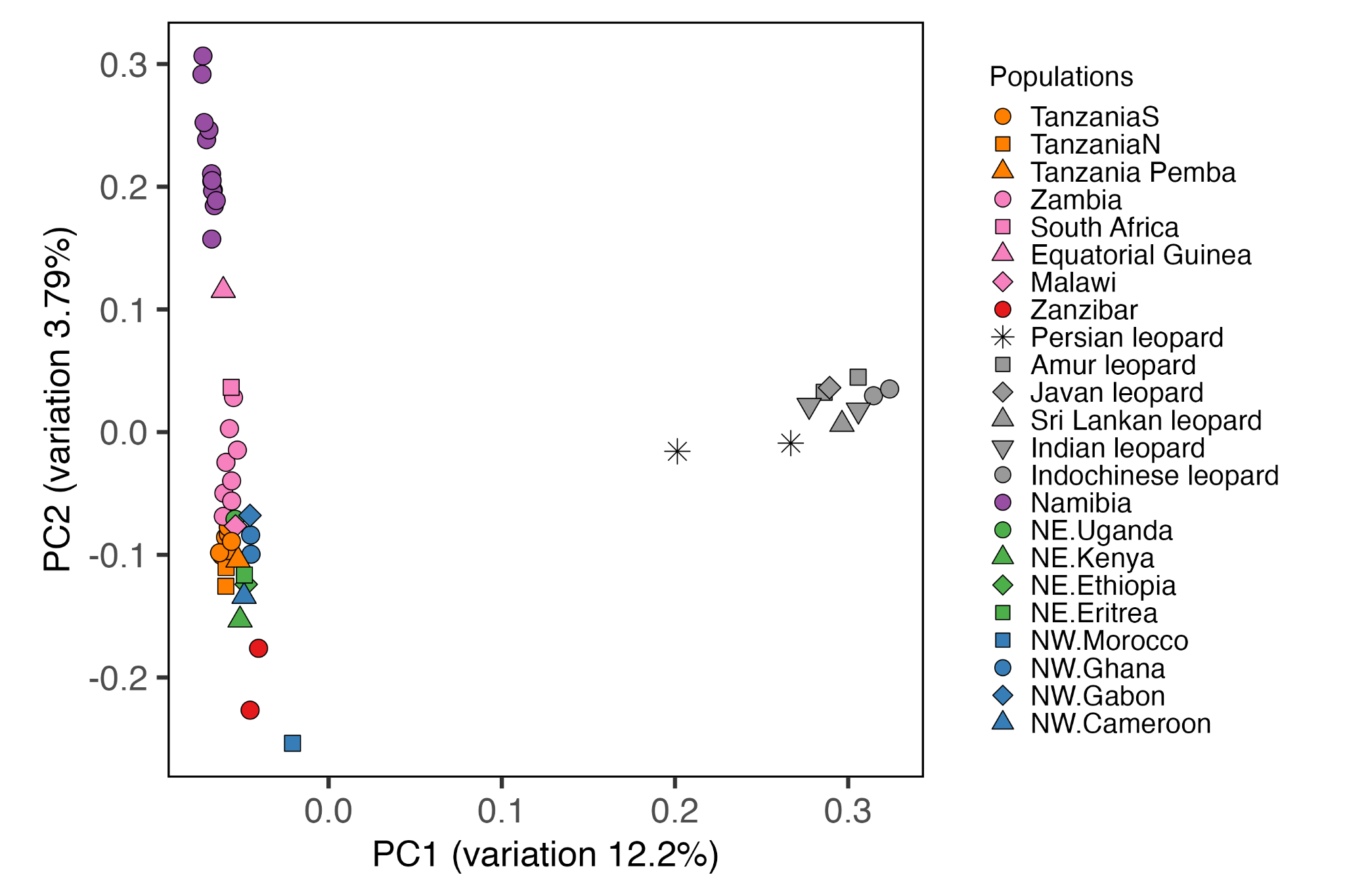


Fig S2. PCA analysis of all leopards included in the dataset (N=60). Icon colours and shapes are labelled according to their geographical origin.


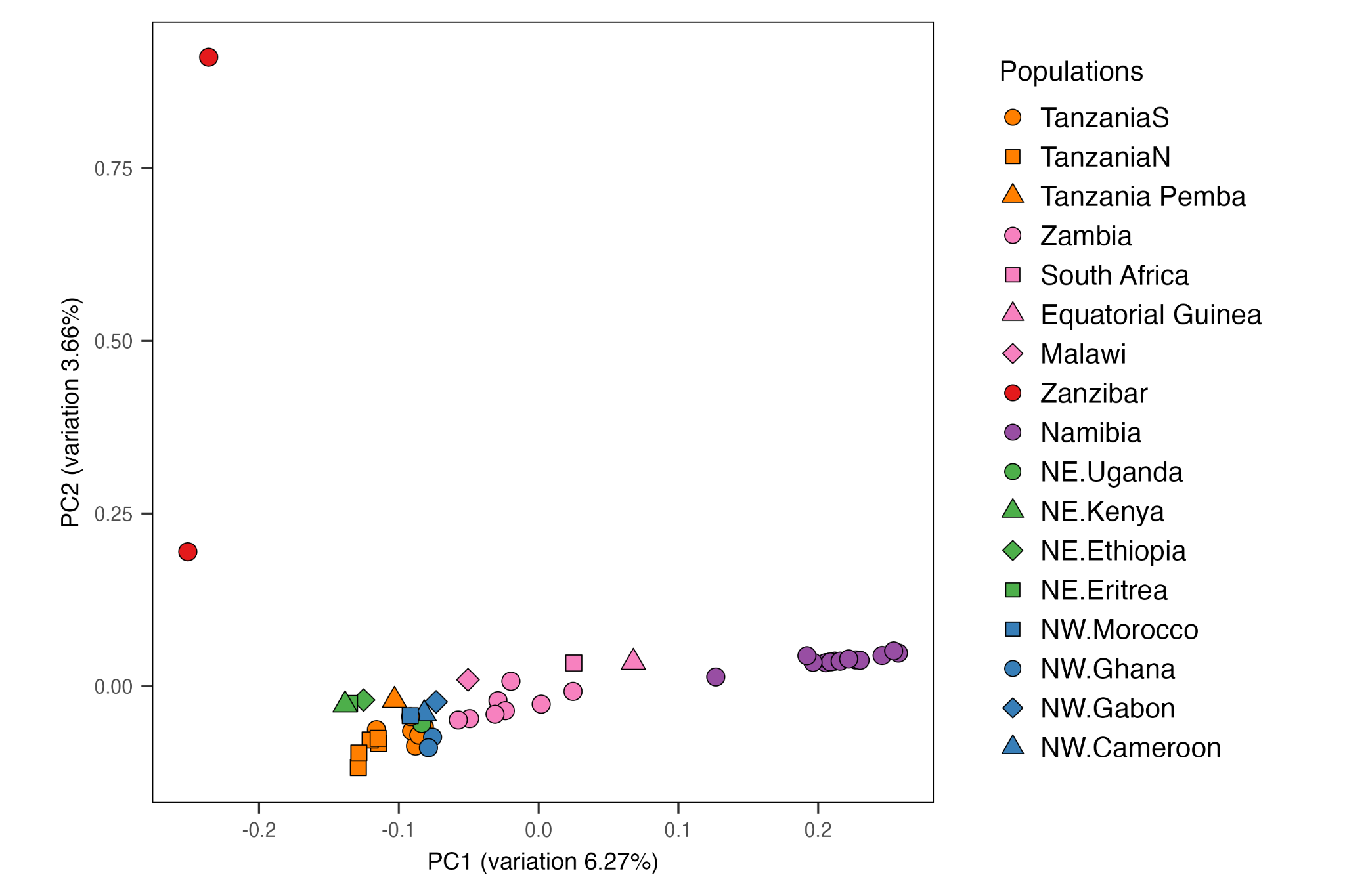


Fig S3. PCA analysis of African leopards included in the dataset. Icon colours and shapes are labelled according to their geographical origin. PC1 and PC2 are shown here.


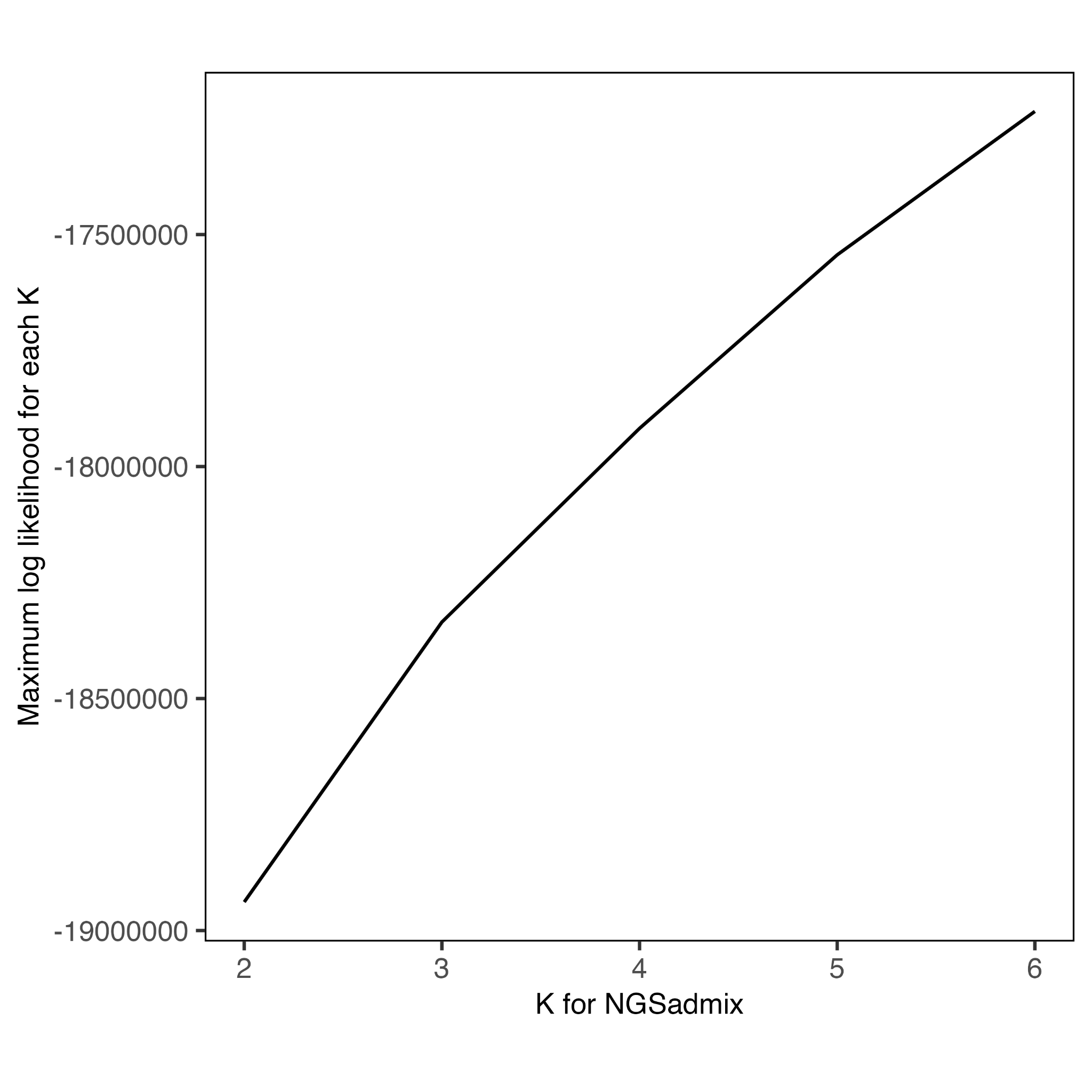


Fig S4. Log-likelihood value of NGSadmix models with different ancestral populations assumed (K=2-6).


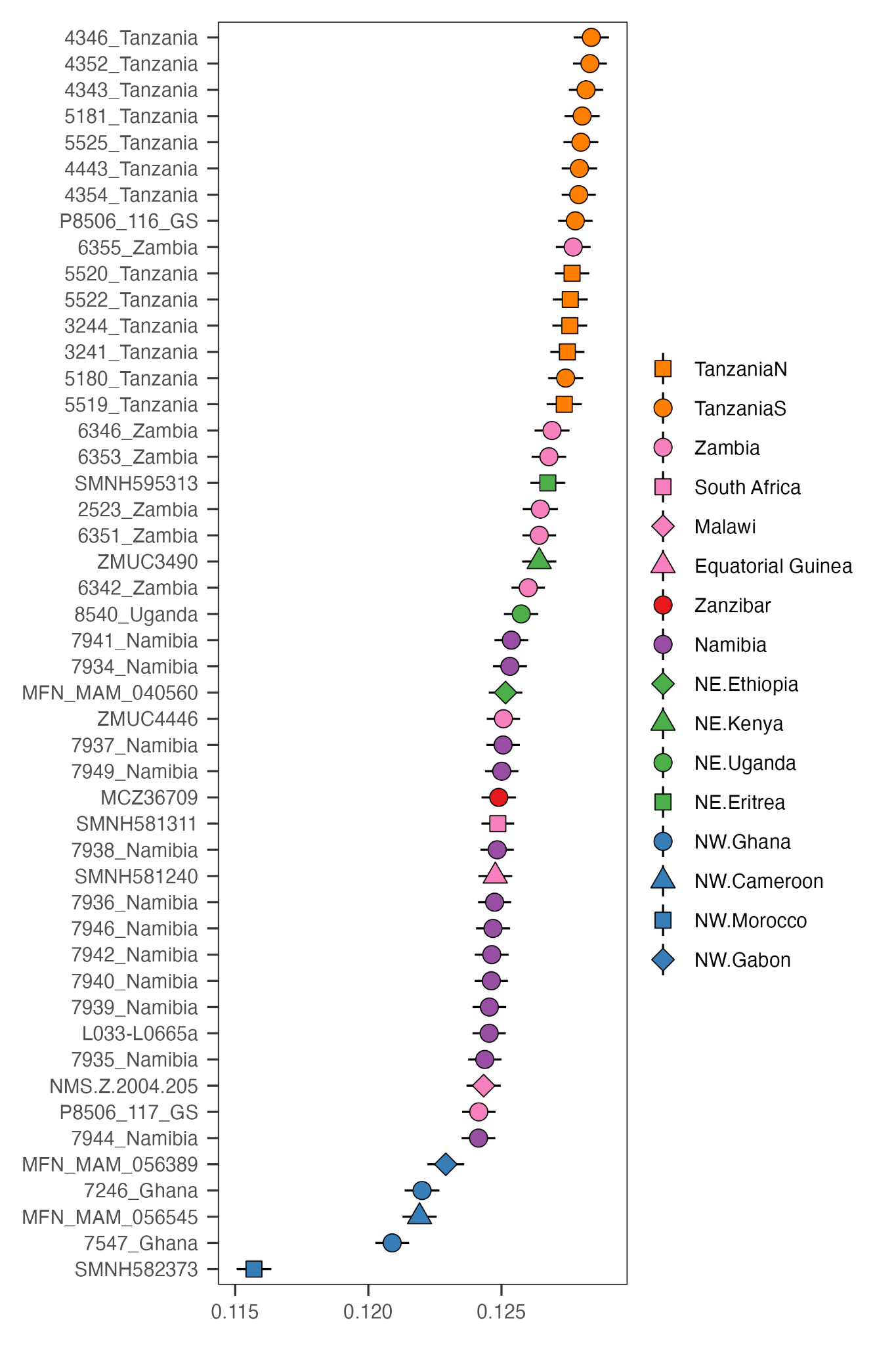


Fig S5. Outgroup f3-statistics comparing shared ancestry between the Pemba Island leopard and mainland African leopards. Icons are coloured and shaped according to their geographical and genetic clusters.


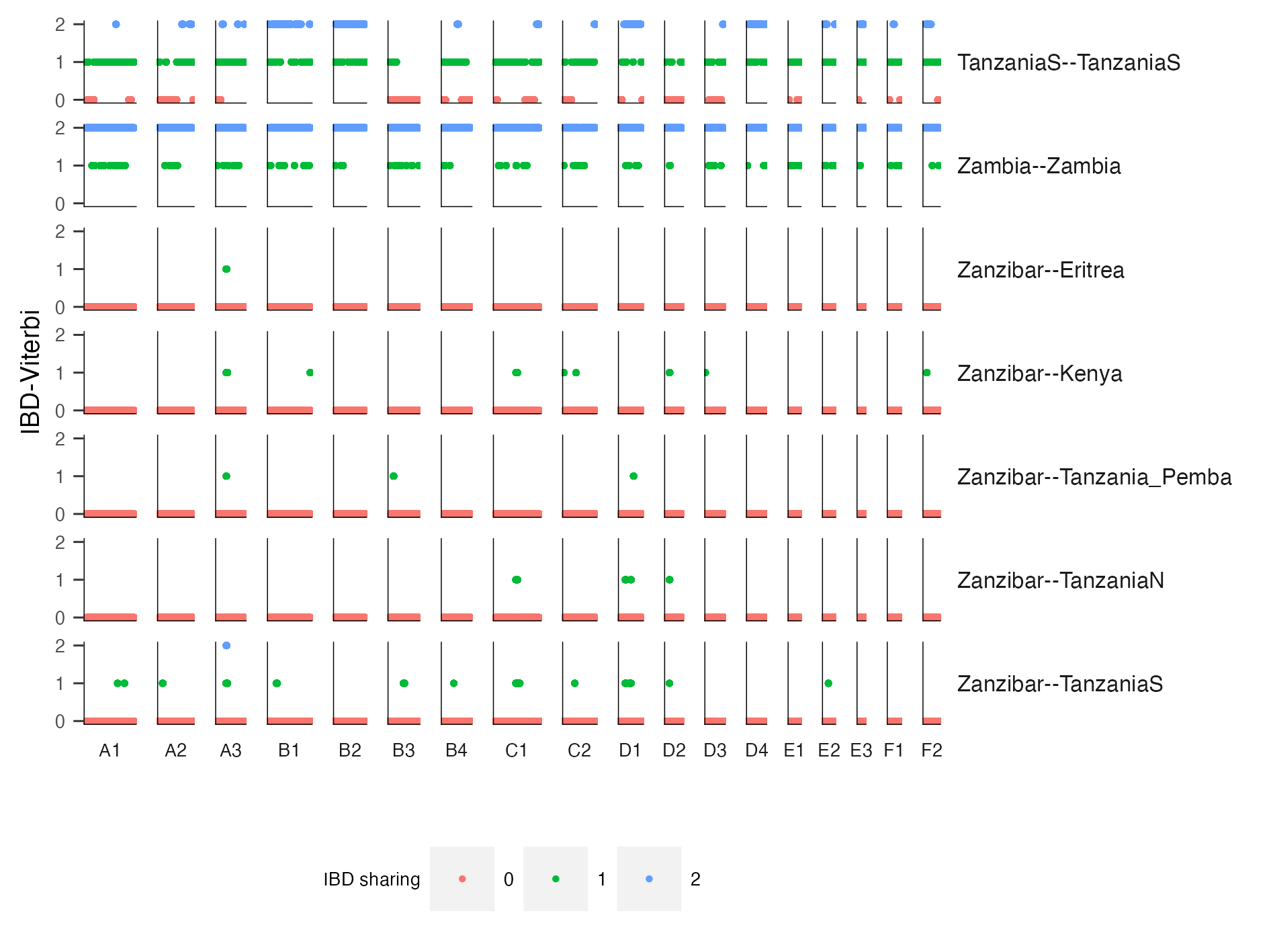


Fig S6. Lack of IBD sharing between Zanzibar leopard and other African leopards. As a comparison, IBD sharing between other African leopards was shown. Y axis refers to the IBD sharing status between the two individuals with 0 as no IBD, 1 as half IBD and 2 as full IBD sharing. X axis refers to chromosomes in the genome.

# Supplementary Tables

Table S1. Sequencing results and other key data for the leopard specimens analysed in this study

Table S2. Gene ontology and KEGG enrichment result of Zanzibar leopard unique homozygous SNPs

Table S3. Candidate gene list for body size in canids and coat colour pattern in domestic cats

Table S4. Body size and coat colour and pattern related genes containing homozygous derived alleles in the Zanzibar leopards
